# 5-Bromo-3-(4-fluoro­phenyl­sulfon­yl)-2-methyl-1-benzofuran

**DOI:** 10.1107/S1600536810027741

**Published:** 2010-07-17

**Authors:** Hong Dae Choi, Pil Ja Seo, Byeng Wha Son, Uk Lee

**Affiliations:** a Department of Chemistry, Dongeui University, San 24 Kaya-dong Busanjin-gu, Busan 614-714, Republic of Korea; b Department of Chemistry, Pukyong National University, 599-1 Daeyeon 3-dong, Nam-gu, Busan 608-737, Republic of Korea

## Abstract

In the title compound, C_15_H_10_BrFO_3_S, the 4-fluoro­phenyl ring makes a dihedral angle of 76.51 (6)° with the plane of the benzofuran fragment. In the crystal, mol­ecules are linked by weak non-classical inter­molecular C—H⋯O hydrogen bonds and an aromatic π–π inter­action between the benzene rings of neighbouring mol­ecules [centroid–centroid distance = 3.540 (3) Å].

## Related literature

For the pharmacological activity of benzofuran compounds, see: Aslam *et al.* (2006 ▶); Galal *et al.* (2009 ▶); Khan *et al.* (2005 ▶). For natural products with benzofuran rings, see: Akgul & Anil (2003 ▶); Soekamto *et al.* (2003 ▶). For the structures of related 3-(4-fluoro­phenyl­sulfon­yl)-2-methyl-1-benzofuran derivatives, see: Choi *et al.* (2010**a* ▶,*b* ▶,c* ▶).
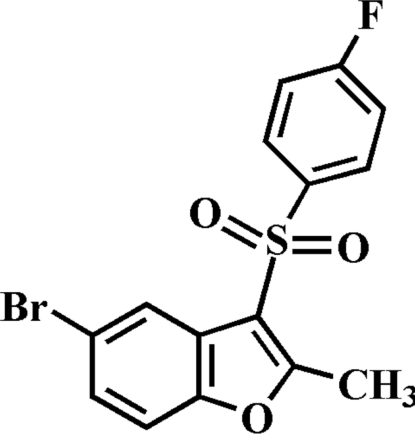


## Experimental

### 

#### Crystal data

C_15_H_10_BrFO_3_S
*M* *_r_* = 369.20
Triclinic, 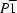

*a* = 7.4519 (3) Å
*b* = 9.2313 (4) Å
*c* = 11.4570 (5) Å
α = 70.652 (2)°
β = 78.495 (2)°
γ = 68.371 (2)°
*V* = 688.57 (5) Å^3^
*Z* = 2
Mo *K*α radiation
μ = 3.15 mm^−1^
*T* = 174 K
0.35 × 0.33 × 0.31 mm

#### Data collection

Bruker SMART APEXII CCD diffractometer
Absorption correction: multi-scan (*SADABS*; Bruker, 2009 ▶) *T*_min_ = 0.405, *T*_max_ = 0.439
11771 measured reflections
3137 independent reflections
2741 reflections with *I* > 2σ(*I*)
*R*_int_ = 0.040

#### Refinement

*R*[*F* ^2^ > 2σ(*F* ^2^)] = 0.030
*wR*(*F* ^2^) = 0.082
*S* = 1.08
3137 reflections
191 parameters
H-atom parameters constrained
Δρ_max_ = 0.23 e Å^−3^
Δρ_min_ = −0.58 e Å^−3^

### 

Data collection: *APEX2* (Bruker, 2009 ▶); cell refinement: *SAINT* (Bruker, 2009 ▶); data reduction: *SAINT*; program(s) used to solve structure: *SHELXS97* (Sheldrick, 2008 ▶); program(s) used to refine structure: *SHELXL97* (Sheldrick, 2008 ▶); molecular graphics: *ORTEP-3* (Farrugia, 1997 ▶) and *DIAMOND* (Brandenburg, 1998 ▶); software used to prepare material for publication: *SHELXL97*.

## Supplementary Material

Crystal structure: contains datablocks global, I. DOI: 10.1107/S1600536810027741/rk2220sup1.cif

Structure factors: contains datablocks I. DOI: 10.1107/S1600536810027741/rk2220Isup2.hkl

Additional supplementary materials:  crystallographic information; 3D view; checkCIF report

## Figures and Tables

**Table 1:** Hydrogen-bond geometry (Å, °)

*D*—H⋯*A*	*D*—H	H⋯*A*	*D*⋯*A*	*D*—H⋯*A*
C15—H15⋯O3^i^	0.95	2.49	3.362 (3)	152
C11—H11⋯O2^ii^	0.95	2.67	3.345 (3)	128

Symmetry codes: (i) 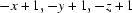; (ii) 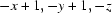.

**Fig. 1.**
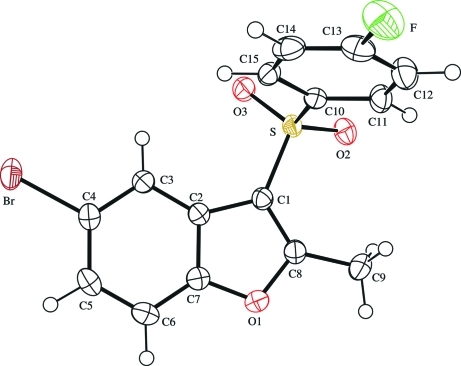
The molecular structure of the title compound with the atom numbering scheme. Displacement ellipsoids are drawn at the 50% probability level. H atoms are presented as a small spheres of arbitrary radius.

**Fig. 2.**
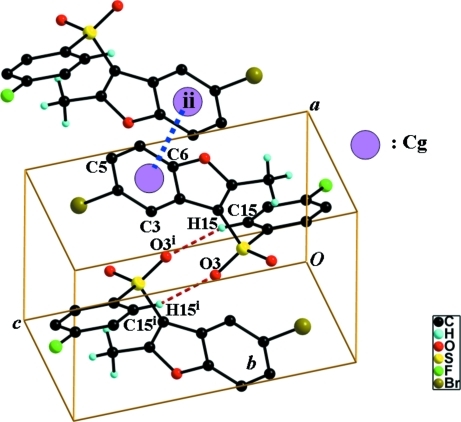
Presentation of non-classical C—H···O and π–π interactions (dashed lines) in the crystal structure of the title compound. Cg denotes the ring centroid. Symmetry codes: (i) -x+1, -y+1, -z+1; (ii) -x+2, -y+1, -z.

## supplementary crystallographic information

### Comment

Many compounds containing a benzofuran moiety show diverse pharmacological properties such as antifungal, antitumor and antiviral, and antimicrobial activities (Aslam *et al.*, 2006, Galal *et al.*, 2009, Khan *et al.*, 2005). These compounds widely occur in nature (Akgul & Anil, 2003; Soekamto *et al.*, 2003). As a part of our study of the substituent effect on the solid state structures of 3-(4-fluorophenylsulfonyl)-2-methyl-1-benzofuran analogues (Choi *et al.*, 2010**a*,*b*,c*), we report the molecular structure of the title compound (Fig. 1).

The benzofuran unit is essentially planar, with a mean deviation of 0.013 (2)Å from the least-squares plane defined by the nine constituent atoms. The dihedral angle formed by the benzofuran plane and the 4-fluorophenyl ring is 76.51 (6)°. The molecular packing (Fig. 2) is stabilized by a weak intermolecular non-classical C—H···O hydrogen bond between the 4-fluorophenyl H atom and the oxygen of the O═S═O unit, with a C15—H15···O3^i^ (Table 1). The crystal packing (Fig. 2) is further stabilized by an aromatic π–π interaction between the benzene rings of neighbouring molecules, with a *Cg*···*Cg*^ii^ distance of 3.540 (3)Å (*Cg* is the centroid of the C2–C7 benzene ring).

### Experimental

The 77% 3-chloroperoxybenzoic acid (493 mg, 2.2 mmol) was added in small portions to a stirred solution of 5-bromo-3-(4-fluorophenylsulfanyl)-2-methyl-1-benzofuran (337 mg, 1.0 mmol) in dichloromethane (40 mL) at 273 K. After being stirred at room temperature for 10 h, the mixture was washed with saturated sodium bicarbonate solution and the organic layer was separated, dried over magnesium sulfate, filtered and concentrated at reduced pressure. The residue was purified by column chromatography (hexane–ethyl acetate, 2:1 v/v) to afford the title compound as a colourless solid [yield 74%, m.p. 459–460 K; *R*_f_=0.68 (hexane–ethyl acetate, 2:1 v/v)]. Single crystals suitable for X-ray diffraction were prepared by slow evaporation of a solution of the title compound in acetone at room temperature.

### Refinement

All H atoms were positioned geometrically and refined using a riding model, with C—H = 0.95Å for aryl and  0.98Å for methyl H atoms. The *U*_iso_(H) = 1.2*U*_eq_(C) for aryl H atoms and *U*_iso_(H) = 1.5*U*_eq_(C) for methyl H atoms.

### Crystal data

**Table:**

C_15_H_10_BrFO_3_S	*Z* = 2
*M**_r_* = 369.20	*F*(000) = 368
Triclinic, *P*1	*D*_x_ = 1.781 Mg m^−^^3^
Hall symbol: -P 1	Mo *K*α radiation, λ = 0.71073 Å
*a* = 7.4519 (3) Å	Cell parameters from 7545 reflections
*b* = 9.2313 (4) Å	θ = 2.5–27.5°
*c* = 11.4570 (5) Å	µ = 3.15 mm^−^^1^
α = 70.652 (2)°	*T* = 174 K
β = 78.495 (2)°	Block, colourless
γ = 68.371 (2)°	0.35 × 0.33 × 0.31 mm
*V* = 688.57 (5) Å^3^	

### Data collection

**Table:**

Bruker SMART APEXII CCD diffractometer	3137 independent reflections
Radiation source: rotating anode	2741 reflections with *I* > 2σ(*I*)
graphite multilayer	*R*_int_ = 0.040
Detector resolution: 10.0 pixels mm^-1^	θ_max_ = 27.5°, θ_min_ = 1.9°
φ– and ω–scans	*h* = −9→9
Absorption correction: multi-scan (*SADABS*; Bruker, 2009)	*k* = −11→11
*T*_min_ = 0.405, *T*_max_ = 0.439	*l* = −14→14
11771 measured reflections	

### Refinement

**Table:**

Refinement on *F*^2^	Primary atom site location: structure-invariant direct methods
Least-squares matrix: full	Secondary atom site location: difference Fourier map
*R*[*F*^2^ > 2σ(*F*^2^)] = 0.030	Hydrogen site location: inferred from neighbouring sites
*wR*(*F*^2^) = 0.082	H-atom parameters constrained
*S* = 1.08	*w* = 1/[σ^2^(*F*_o_^2^) + (0.0442*P*)^2^ + 0.0824*P*] where *P* = (*F*_o_^2^ + 2*F*_c_^2^)/3
3137 reflections	(Δ/σ)_max_ = 0.001
191 parameters	Δρ_max_ = 0.23 e Å^−^^3^
0 restraints	Δρ_min_ = −0.58 e Å^−^^3^

### Special details

**Table:**

Geometry. All s.u.'s (except the s.u. in the dihedral angle between two l.s. planes) are estimated using the full covariance matrix. The cell s.u.'s are taken into account individually in the estimation of s.u.'s in distances, angles and torsion angles; correlations between s.u.'s in cell parameters are only used when they are defined by crystal symmetry. An approximate (isotropic) treatment of cell s.u.'s is used for estimating s.u.'s involving l.s. planes.
Refinement. Refinement of *F*^2^ against ALL reflections. The weighted *R*-factor *wR* and goodness of fit *S* are based on *F*^2^, conventional *R*-factors *R* are based on *F*, with *F* set to zero for negative *F*^2^. The threshold expression of *F*^2^ > 2σ(*F*^2^) is used only for calculating *R*-factors(gt) etc. and is not relevant to the choice of reflections for refinement. *R*-factors based on *F*^2^ are statistically about twice as large as those based on *F*, and *R*-factors based on ALL data will be even larger.

### Fractional atomic coordinates and isotropic or equivalent isotropic displacement parameters (Å^2^)

**Table:**

	*x*	*y*	*z*	*U*_iso_*/*U*_eq_	
Br	0.75115 (3)	0.08439 (3)	0.82425 (2)	0.04195 (10)	
S	0.46680 (7)	0.39556 (6)	0.29542 (5)	0.02422 (12)	
F	1.0254 (2)	0.73795 (18)	0.06607 (16)	0.0525 (4)	
O1	0.7799 (2)	−0.06606 (16)	0.35232 (13)	0.0283 (3)	
O2	0.3581 (2)	0.41371 (18)	0.19806 (14)	0.0315 (3)	
O3	0.3671 (2)	0.44837 (18)	0.40280 (14)	0.0331 (3)	
C1	0.6054 (3)	0.1915 (2)	0.34920 (18)	0.0244 (4)	
C2	0.6740 (3)	0.1097 (2)	0.47105 (18)	0.0229 (4)	
C3	0.6615 (3)	0.1518 (2)	0.57888 (18)	0.0258 (4)	
H3	0.5897	0.2583	0.5852	0.031*	
C4	0.7587 (3)	0.0310 (3)	0.67691 (19)	0.0273 (4)	
C5	0.8637 (3)	−0.1268 (3)	0.6709 (2)	0.0304 (5)	
H5	0.9269	−0.2057	0.7407	0.037*	
C6	0.8759 (3)	−0.1687 (2)	0.5639 (2)	0.0293 (5)	
H6	0.9468	−0.2754	0.5577	0.035*	
C7	0.7807 (3)	−0.0488 (2)	0.46705 (19)	0.0249 (4)	
C8	0.6742 (3)	0.0817 (2)	0.28167 (19)	0.0262 (4)	
C9	0.6637 (3)	0.0903 (3)	0.1523 (2)	0.0356 (5)	
H9A	0.5777	0.1978	0.1109	0.053*	
H9B	0.6128	0.0067	0.1516	0.053*	
H9C	0.7936	0.0722	0.1083	0.053*	
C10	0.6374 (3)	0.4971 (2)	0.22804 (19)	0.0240 (4)	
C11	0.6702 (3)	0.5460 (3)	0.0994 (2)	0.0305 (5)	
H11	0.6034	0.5226	0.0491	0.037*	
C12	0.8000 (3)	0.6283 (3)	0.0455 (2)	0.0373 (5)	
H12	0.8227	0.6642	−0.0423	0.045*	
C13	0.8962 (3)	0.6577 (3)	0.1208 (2)	0.0354 (5)	
C14	0.8684 (3)	0.6105 (3)	0.2478 (2)	0.0343 (5)	
H14	0.9387	0.6324	0.2969	0.041*	
C15	0.7348 (3)	0.5297 (2)	0.3030 (2)	0.0283 (4)	
H15	0.7103	0.4971	0.3908	0.034*	

### Atomic displacement parameters (Å^2^)

**Table:**

	*U*^11^	*U*^22^	*U*^33^	*U*^12^	*U*^13^	*U*^23^
Br	0.04901 (16)	0.04935 (17)	0.02543 (14)	−0.00911 (12)	−0.01044 (10)	−0.01172 (11)
S	0.0240 (2)	0.0251 (3)	0.0201 (2)	−0.00436 (19)	−0.00352 (19)	−0.0053 (2)
F	0.0530 (9)	0.0482 (9)	0.0613 (10)	−0.0315 (7)	−0.0082 (7)	−0.0033 (8)
O1	0.0304 (7)	0.0261 (7)	0.0282 (8)	−0.0070 (6)	−0.0013 (6)	−0.0107 (6)
O2	0.0301 (7)	0.0349 (8)	0.0286 (8)	−0.0102 (6)	−0.0104 (6)	−0.0038 (7)
O3	0.0335 (8)	0.0316 (8)	0.0249 (8)	−0.0015 (6)	0.0021 (6)	−0.0091 (7)
C1	0.0239 (9)	0.0257 (10)	0.0216 (10)	−0.0067 (8)	−0.0014 (8)	−0.0060 (8)
C2	0.0202 (8)	0.0238 (10)	0.0231 (10)	−0.0077 (7)	−0.0004 (7)	−0.0050 (8)
C3	0.0264 (9)	0.0249 (10)	0.0232 (10)	−0.0062 (8)	−0.0019 (8)	−0.0058 (8)
C4	0.0239 (9)	0.0341 (11)	0.0226 (10)	−0.0098 (8)	−0.0026 (8)	−0.0060 (9)
C5	0.0269 (10)	0.0305 (11)	0.0276 (11)	−0.0086 (8)	−0.0053 (8)	0.0006 (9)
C6	0.0242 (9)	0.0231 (10)	0.0344 (12)	−0.0048 (8)	−0.0012 (9)	−0.0044 (9)
C7	0.0240 (9)	0.0260 (10)	0.0256 (10)	−0.0102 (8)	0.0003 (8)	−0.0076 (8)
C8	0.0254 (9)	0.0277 (10)	0.0241 (10)	−0.0078 (8)	−0.0022 (8)	−0.0064 (8)
C9	0.0418 (12)	0.0394 (13)	0.0278 (12)	−0.0100 (10)	−0.0040 (10)	−0.0154 (10)
C10	0.0256 (9)	0.0202 (9)	0.0245 (10)	−0.0037 (7)	−0.0048 (8)	−0.0069 (8)
C11	0.0368 (11)	0.0335 (11)	0.0242 (10)	−0.0135 (9)	−0.0076 (9)	−0.0070 (9)
C12	0.0432 (13)	0.0379 (13)	0.0295 (12)	−0.0179 (10)	−0.0021 (10)	−0.0036 (10)
C13	0.0363 (11)	0.0255 (11)	0.0456 (14)	−0.0126 (9)	−0.0061 (10)	−0.0072 (10)
C14	0.0358 (11)	0.0271 (11)	0.0459 (14)	−0.0074 (9)	−0.0137 (10)	−0.0153 (10)
C15	0.0323 (10)	0.0248 (10)	0.0253 (10)	−0.0005 (8)	−0.0084 (8)	−0.0105 (9)

### Geometric parameters (Å, °)

**Table:**

Br—C4	1.894 (2)	C6—C7	1.371 (3)
S—O2	1.4369 (15)	C6—H6	0.9500
S—O3	1.4385 (14)	C8—C9	1.474 (3)
S—C1	1.745 (2)	C9—H9A	0.9800
S—C10	1.760 (2)	C9—H9B	0.9800
F—C13	1.359 (3)	C9—H9C	0.9800
O1—C8	1.366 (2)	C10—C11	1.390 (3)
O1—C7	1.377 (2)	C10—C15	1.391 (3)
C1—C8	1.367 (3)	C11—C12	1.374 (3)
C1—C2	1.443 (3)	C11—H11	0.9500
C2—C3	1.390 (3)	C12—C13	1.370 (3)
C2—C7	1.396 (3)	C12—H12	0.9500
C3—C4	1.389 (3)	C13—C14	1.370 (3)
C3—H3	0.9500	C14—C15	1.390 (3)
C4—C5	1.394 (3)	C14—H14	0.9500
C5—C6	1.380 (3)	C15—H15	0.9500
C5—H5	0.9500		
			
O2—S—O3	119.74 (9)	O1—C8—C1	110.28 (18)
O2—S—C1	108.91 (9)	O1—C8—C9	115.36 (17)
O3—S—C1	106.96 (9)	C1—C8—C9	134.3 (2)
O2—S—C10	107.49 (9)	C8—C9—H9A	109.5
O3—S—C10	107.99 (10)	C8—C9—H9B	109.5
C1—S—C10	104.78 (9)	H9A—C9—H9B	109.5
C8—O1—C7	107.04 (14)	C8—C9—H9C	109.5
C8—C1—C2	107.63 (17)	H9A—C9—H9C	109.5
C8—C1—S	125.95 (17)	H9B—C9—H9C	109.5
C2—C1—S	126.38 (15)	C11—C10—C15	120.92 (19)
C3—C2—C7	119.08 (18)	C11—C10—S	118.93 (16)
C3—C2—C1	136.46 (18)	C15—C10—S	120.14 (16)
C7—C2—C1	104.44 (17)	C12—C11—C10	119.6 (2)
C4—C3—C2	116.97 (18)	C12—C11—H11	120.2
C4—C3—H3	121.5	C10—C11—H11	120.2
C2—C3—H3	121.5	C13—C12—C11	118.7 (2)
C3—C4—C5	122.9 (2)	C13—C12—H12	120.7
C3—C4—Br	118.43 (15)	C11—C12—H12	120.7
C5—C4—Br	118.66 (16)	F—C13—C14	118.7 (2)
C6—C5—C4	120.1 (2)	F—C13—C12	118.0 (2)
C6—C5—H5	119.9	C14—C13—C12	123.4 (2)
C4—C5—H5	119.9	C13—C14—C15	118.3 (2)
C7—C6—C5	116.77 (18)	C13—C14—H14	120.9
C7—C6—H6	121.6	C15—C14—H14	120.9
C5—C6—H6	121.6	C14—C15—C10	119.2 (2)
C6—C7—O1	125.27 (18)	C14—C15—H15	120.4
C6—C7—C2	124.12 (19)	C10—C15—H15	120.4
O1—C7—C2	110.59 (17)		
			
O2—S—C1—C8	28.0 (2)	C1—C2—C7—O1	0.0 (2)
O3—S—C1—C8	158.69 (18)	C7—O1—C8—C1	−1.0 (2)
C10—S—C1—C8	−86.8 (2)	C7—O1—C8—C9	177.27 (18)
O2—S—C1—C2	−154.43 (17)	C2—C1—C8—O1	0.9 (2)
O3—S—C1—C2	−23.7 (2)	S—C1—C8—O1	178.94 (14)
C10—S—C1—C2	90.80 (19)	C2—C1—C8—C9	−176.8 (2)
C8—C1—C2—C3	177.7 (2)	S—C1—C8—C9	1.2 (4)
S—C1—C2—C3	−0.3 (3)	O2—S—C10—C11	−12.64 (18)
C8—C1—C2—C7	−0.6 (2)	O3—S—C10—C11	−143.12 (15)
S—C1—C2—C7	−178.53 (15)	C1—S—C10—C11	103.12 (17)
C7—C2—C3—C4	0.3 (3)	O2—S—C10—C15	166.35 (15)
C1—C2—C3—C4	−177.8 (2)	O3—S—C10—C15	35.87 (18)
C2—C3—C4—C5	−0.6 (3)	C1—S—C10—C15	−77.89 (17)
C2—C3—C4—Br	178.41 (14)	C15—C10—C11—C12	−0.3 (3)
C3—C4—C5—C6	0.6 (3)	S—C10—C11—C12	178.67 (17)
Br—C4—C5—C6	−178.47 (15)	C10—C11—C12—C13	1.0 (3)
C4—C5—C6—C7	−0.1 (3)	C11—C12—C13—F	179.4 (2)
C5—C6—C7—O1	178.37 (18)	C11—C12—C13—C14	−0.6 (4)
C5—C6—C7—C2	−0.2 (3)	F—C13—C14—C15	179.46 (19)
C8—O1—C7—C6	−178.15 (19)	C12—C13—C14—C15	−0.5 (3)
C8—O1—C7—C2	0.6 (2)	C13—C14—C15—C10	1.2 (3)
C3—C2—C7—C6	0.1 (3)	C11—C10—C15—C14	−0.8 (3)
C1—C2—C7—C6	178.73 (19)	S—C10—C15—C14	−179.79 (15)
C3—C2—C7—O1	−178.63 (16)		

### Hydrogen-bond geometry (Å, °)

**Table:**

*D*—H···*A*	*D*—H	H···*A*	*D*···*A*	*D*—H···*A*
C15—H15···O3^i^	0.95	2.49	3.362 (3)	152
C11—H11···O2^ii^	0.95	2.67	3.345 (3)	128

Symmetry codes: (i) −*x*+1, −*y*+1, −*z*+1; (ii) −*x*+1, −*y*+1, −*z*.
